# Activity in the dorsal ACC causes deterioration of sequential motor performance due to anxiety

**DOI:** 10.1038/s41467-019-12205-6

**Published:** 2019-09-19

**Authors:** Gowrishankar Ganesh, Takehiro Minamoto, Masahiko Haruno

**Affiliations:** 1 Centre National de la Recherche Scientifique (CNRS)—Universite Montpellier (UM) Laboratoire d’Informatique, de Robotique et de Microelectronique de Montpellier (LIRMM), Montpellier, France; 2 CNRS-National Institute of Advanced Industrial Science and Technology (AIST) Joint Robotics Laboratory (JRL), Tsukuba, Japan; 3 Faculty of Human Sciences, Shimane University, Matsue, Japan; 4 Center for Information and Neural Networks, National Institute of Information and Communications Technology (NICT), Osaka, Japan; 5 Graduate School of Frontier Biosciences, Osaka University, Osaka, Japan

**Keywords:** Functional magnetic resonance imaging, Cognitive control, Emotion, Sensorimotor processing

## Abstract

Performance anxiety can profoundly affect motor performance, even in experts such as professional athletes and musicians. Previously, the neural mechanisms underlying anxiety-induced performance deterioration have predominantly been investigated for individual one-shot actions. Sports and music, however, are characterized by action sequences, where many individual actions are assembled to develop a performance. Here, utilizing a novel differential sequential motor learning paradigm, we first show that performance at the junctions between pre-learnt action sequences is particularly prone to anxiety. Next, utilizing functional magnetic resonance imaging (fMRI), we reveal that performance deterioration at the junctions is parametrically correlated with activity in the dorsal anterior cingulate cortex (dACC). Finally, we show that 1 Hz repetitive transcranial magnetic stimulation of the dACC attenuates the performance deterioration at the junctions. These results demonstrate causality between dACC activity and impairment of sequential motor performance due to anxiety, and suggest new intervention techniques against the deterioration.

## Abstract (web summary)

Performance anxiety can impair motor skill, and even affect expert athletes and musicians. Here, the authors show that anxiety affects performance at the ‘junction’ between two well-learned action sequences, and that this affect is associated with activity in dorsal anterior cingulate cortex (dACC).

## Introduction

Performance anxiety is a key issue in cognitive and sports sciences^1–5^, and several brain imaging studies have investigated the neural mechanisms underlying the relation between motor performance and reward processing^6–10^. However, the neural mechanisms have been predominantly investigated for tasks requiring single, one-shot actions like generating a grip force^8–10^ or reaching^6^. Sports and music performances, on the other hand, are characterized by a sequential assembly of pre-learnt actions. For instance, tennis players learn individual motor actions like a serve, return, and volley separately, and have to assemble these components into a sequence in a match under the influence of anxiety. This assembly of actions is believed to develop *explicitly*, guided by cognitive action choices at the initial stages of the performance (sports or music) training and become increasingly automatic with an individual’s experience^11,12^. Interestingly, the popular, so called self-focus theories of performance anxiety^1–3^ propose that performance deterioration is caused by re-activation of the explicit choice processes and their subsequent interference with automatic control in the presence of anxiety. Together, these two theories about sequence and explicit choice suggest that, in addition to the effect on individual actions^6–10^, anxiety should also affect the cognition and control of the assembled actions in sequential motor tasks.

Medial prefrontal cortices, especially the dorsal anterior cingulate cortex (dACC), are well known for their role in cognitive controls during sequential or hierarchical decision-making^13–16^. Furthermore, the dACC is known to be involved in the processing of anxiety^17,18^. In fact, a previous study that analyzed the effect of anxiety in a target pursuit task^7^, which can be considered a sequential task requiring the assembly of different movement directions, observed activations in the medial prefrontal cortex correlated with the performance modulation by reward. Based on these observations, we expected a critical role of the dACC in the deterioration of sequential motor performance due to anxiety (DSMPA), but to confirm this we first needed a suitable task.

**Fig. 1:**
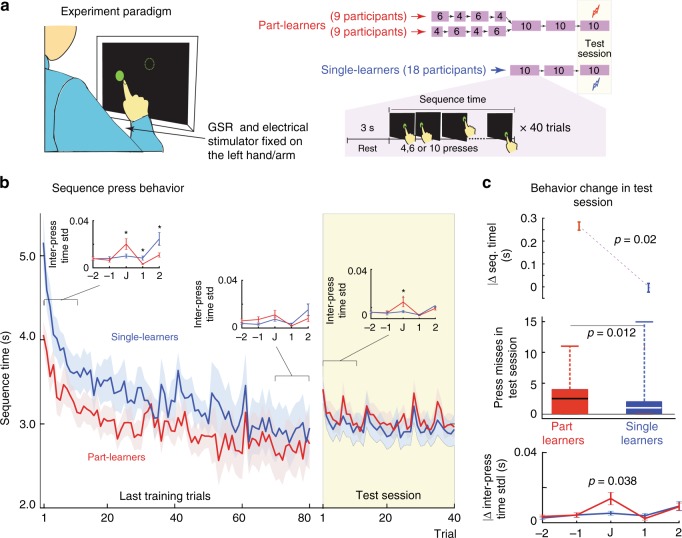
Experiment 1. **a** The participants learnt to press a sequence of circles that appeared one after another on a computer screen in front of them. As part learners, they trained on the first six or four circles in a sequence before training on the remaining circles (see right panel). They then went on to train on the whole 10-circle sequence and finally took an anxiety test on the sequence. Single learners trained only on the whole 10-circle sequence before taking the anxiety test. All sessions included 40 sequence trials. **b** The press sequence time through the last training trials (white background) and the anxiety test session (yellow background) for part learners (red trace) and single learners (blue trace). The shaded area represents the across participant standard error. The inset figures show the local variation in the standard deviation in the inter-press time aligned with the junction “J” (either after the fourth or sixth circle in **a**) of the pre-learnt sequences by the part learners. For single learners, J was chosen either after the fourth or sixth circle randomly. Asterisk show a difference in the inset figures that is significant (*p* < 0.05). **c** The change of participant behavior in the anxiety test session. The across participant change in the sequence press time is shown in the top panel. The average number of times participants pressed a wrong circle and received an electrical shock in a test session are shown in the middle panel. On each box of the boxplot, the line within the box shows the median, the edges of the box are the 25th and 75th percentiles of the data, and the whiskers show the range of the data points. The change in the inter-press time standard deviation (STD) between the first 20 test trials and the last training session is shown in bottom panel. Error bars in the figure represent standard error. All *p* values represent paired one-sample tests. We checked for the normality of the datasets using the Shapiro–Wilk test before each comparison. To get the *p*-values a *t*-test was used for comparisons when the data groups were normal, and a Wilcoxon signed rank test were used when one or both of the datasets were non-normal (see text for details)

**Fig. 2:**
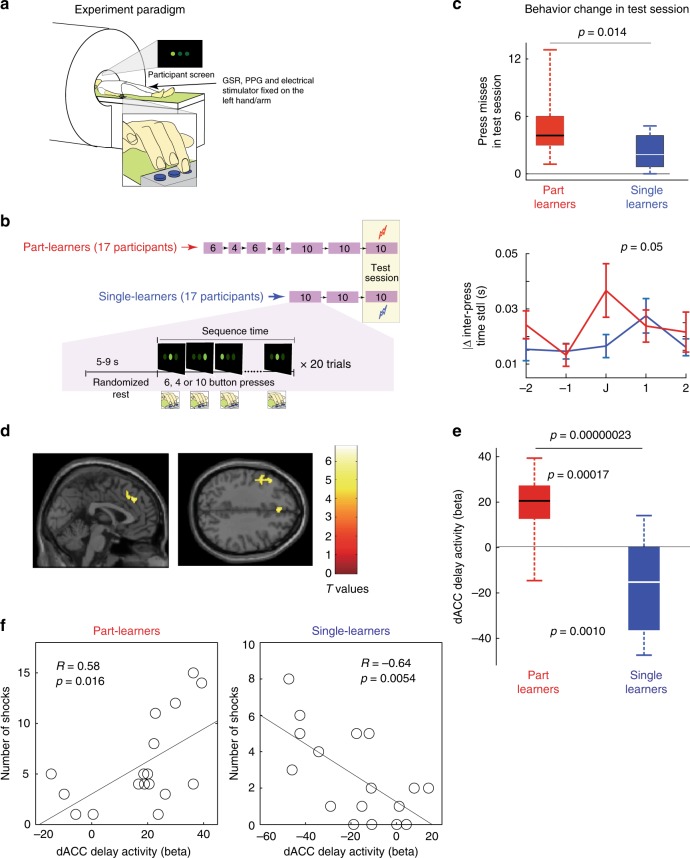
Experiment 2: **a** The participants lay in an fMRI scanner and performed a sequential button-press task in which they pressed three buttons in a sequence guided by a visual cue on the screen above them. **b** Each participant worked as either a part learner or single learner in sessions of 20 trials as shown. **c** The number of press misses are shown in the top panel with boxplots. The bottom panel shows the absolute change in the inter-press time standard deviation (STD) near the junction in the anxiety test session. Error bars show standard error. **d** A parametric event-related general linear model analysis was conducted in which the seventh button in every trial was defined as the junction event, and the inter-press time in every trial at the junction was used as the parametric regressor and contrasted between the part- and single learners. A two-sample *t*-test demonstrated that activity in the dACC (peak MNI coordinates, [0, 36, 36]) and left and dorsal premotor cortices (peak MNI coordinates, [−48, 8, 42]) was significantly higher in part learners than in single learners (*p* < 0.05, family-wise-error (FWE) corrected). We used *p* < 0.0005 uncorrected for display purposes here. **e** The beta values for part learners were positive (*p* = 0.00017, one-sample *t*-test), while those for single learners were negative (*p* = 0.00010, one-sample *t*-test), and these values were significantly different (*p* = 0.00000023, two-sample *t*-test). **f** The relationship between differential brain activity and the number of electrical shocks. dACC activity was positively correlated with the number of electrical shocks in part learners (left, *R* = 0.58, *p* = 0.016), while the correlation was negative in single learners (right, *R* = −0.64, *p* = 0.0054). No correlation with shocks was observed in the left premotor cortex of the same part learners (*R* = 0.38 and *p* = 0.14) or single learners (*R* = 0.11 and *p* = 0.67). On the boxplots in **c** and **e**, the line within the box shows the median, the edges of the box are the 25th and 75th percentiles of the data, and the whiskers show the range of the data points

**Fig. 3:**
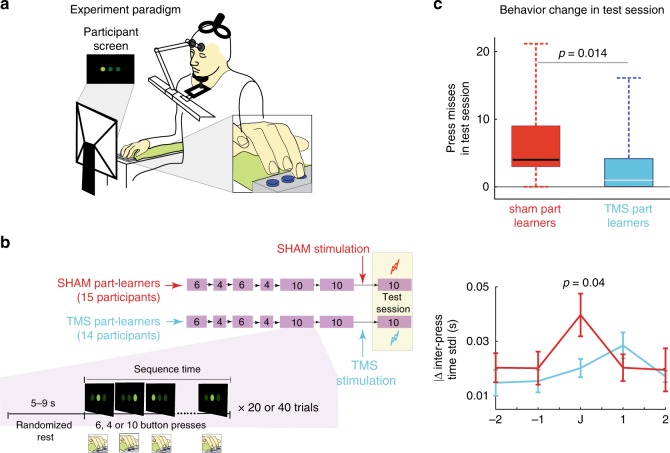
Experiment 3: **a** All participants participated as part learners. They pressed a button pad as in Experiment 2, but in a sitting position. **b** They trained on the first six and then next four buttons in the sequence, followed by two training sessions on the entire 10-button sequence, again as in Experiment 2. Half of the participants (TMS part learners) then were stimulated with rTMS to their dACC, and the other half (SHAM part learners) experienced SHAM stimulations for the same period. **c** The number of press misses are shown in the top panel with boxplots for the SHAM part learners (red plot) and TMS part learners (cyan data). On each box of the boxplot, the line within the box shows the median, the edges of the box are the 25th and 75th percentiles of the data, and the whiskers show the range of the data points. The bottom panel shows the absolute change in the inter-press time standard deviation (STD) near the junction in the anxiety test session. Error bars show standard error. The similarity of plots in Figs. 2c and 3c shows rTMS to the dACC attenuates the effects of anxiety on part learners, making them similar to single learners. All error bars represent standard error. All *p* values represent two-sample tests. We checked for the normality of the datasets using the Shapiro–Wilk test before each comparison. To get the *p* values, a *t*-test was used for comparisons when the data groups were normal, and a Wilcoxon rank-sum test were used when one or both of the datasets were non-normal (see text for details)

We thus started by designing a novel *differential* motor sequence learning (DMSL) task. Our DMSL task required participants to learn, through repeated trials, to perform a given sequence of ten button presses as fast as possible with their fingers. We divided the participants into two groups. *Single learners* trained directly on a 10-button sequence and *part learners* pre-trained on the first six and then remaining four presses (or the first four and then remaining six presses) of the 10-button sequence before going on to train on the complete 10-button sequence for the same time as the single learners (see Figs. 1a, 2b and 3b). After the training, both groups were subjected to an anxiety *test session* on the trained 10-button sequence, in which the participants suffered an electrical shock on their arm if they made an error in the button sequence or were *too slow* in their pressing task.

Previous studies have shown that the motor deterioration during anxiety is greater when the initial motor learning is accompanied by more cognitive processes^2,3^. Self-focus theories of performance anxiety^1–3^ suggest that in the presence of anxiety, the initial cognitive processes are reactivated, leading to evaluative considerations about self-generated actions, which then interfere with the performance. Consequently, we made two behavioral hypotheses and one neural hypothesis for our DMSL task. First, we hypothesized that part learners would explicitly combine the pre-learnt parts when they perform the 10-button sequence, leading to an increase in the utilized cognitive processes compared to single learners. Thus, while the pre-training may help part learners perform the 10-button sequence better than single learners, it should cause a larger deterioration in their performance in the anxiety test session^19^. In other words, we hypothesized that our task will enable us to modulate DSMPA through differential learning strategies. Second, we hypothesized that, because part learners join two pre-learnt sequences, the cognitive processes and hence the consequent motor deterioration are predominantly localized at the *junction* of the pre-learnt parts. And finally, in line with these behavioral hypotheses, considering that the dACC has been previously implicated in cognitive control^13–16^ and exploratory action selection^15,20,21^, we made the neural hypothesis that the dACC activity differentially determines the deterioration in single learners and part learners.

We utilize three experiments to show the validity of these hypotheses. Using a behavioral experiment, we first show that DSMPA can be modulated by a DMSL task and that the performance at the junctions between pre-learnt actions is particularly prone to deterioration due to anxiety. Using fMRI of the DMSL task, we then show that the performance deterioration is parametrically correlated with activity in the dACC. Finally, to exhibit causality between the dACC activity and the deterioration, we show that the suppression of dACC activity using 1 Hz repetitive transcranial magnetic stimulation attenuates the performance deterioration at the junctions.

## Results

### Experiment 1

We started with the test of the two behavioral hypotheses in Experiment 1 (Fig. 1), which included 18 right-handed participants. The participants sat in front of a touch screen (Fig. 1a) and were presented with a sequence of green circles on the screen as “buttons”, one at a time, which they pressed/touched with the index finger of their right hand. Each correct press extinguished the touched button and presented the next button in a sequence. The participants worked in repeated training trials of each sequence and were instructed to “focus your training on speeding up your press sequence as much as possible and make sure you don’t miss the buttons”. We utilized two distinct spatial button sequences in this experiment, and every participant trained on both of them as a single learner in one sequence and part learner in the other. The order of the single/part learning sessions and the sequence utilized for each were balanced across participants.

As a part learner, participants pre-learnt parts of a 10-button sequence. One half of them trained on the first six buttons and then the next four buttons of the sequence in separate preliminary sessions, and the other half trained on the first four buttons and the next six buttons of the sequence similarly. We utilized the two different patterns for the part learners in order to negate effects of a specific chunk pattern^22,23^. The participants then went on to train on the complete 10-button sequence session (Fig. 1a). As single learners, participants directly trained on the 10-button sequence. Both single- and part learners trained for the same number of trials on the complete 10-button sequence and immediately after this training, took part in an anxiety test session of the sequence they had learnt. The participants were told that “you will suffer an electric shock if you miss any button. You are allowed to slow your presses, but if it becomes too slow and below a “time threshold”, you will suffer a shock at the end of the trial”. The electric shock was provided through electrodes fixed to the forearm of their left arm. The electrical shock was calibrated for each participant (see Methods for details) and was demonstrated to the participants before the start of the experiment. Unknown to the participants, the “time threshold” was tuned for each participant and set to 1.5 times their average sequence time of their last ten training trials.

We first quantified the participant’s behavior by their *sequence time*; the time taken for completion of the 10 button-press sequence (Fig. 1b). We observed that part learners were consistently faster in the task compared to single learners. A two-way ANOVA across trials and learners showed a significant main effect of trials (*F*(79, 2171) = 4.28, *p* < 0.0001) and learning type (*F*(1, 2171) = 86.69, *p* < 0.00001), but no interaction (*F*(79, 2171) = 0.39, *p* = 0.39). Part learners showed significantly faster sequence time in the last 15 trials compared to single learners (*T*(17) = 2.44, *p* = 0.026, one-sample *t*-test on the average sequence time in the last 15 trials).

Starting the test sessions increased the anxiety in the participants. The galvanic skin response (GSR) was observed to be significantly higher in the start of the test session, compared to the start of the last training session both in single learners (*T*(17) = 2.17, *p* = 0.04, one-sample *t*-test) and part learners (*T*(17) = 2.35, *p* = 0.03, one-sample *t*-test). There was no difference in GSR changes between the single and part learners (*T*(17) = 0.09, *p* = 0.93, one-sample *t*-test). As mentioned before, the part learners performed better at the end of the training session compared to the single learners. However, in the anxiety test session, the behaviors flipped, with part learners slower than single learners. Overall, the slowing of part learners was significantly more than of single learners (top panel, Fig. 1c; *Z* = 2.32, signed rank = 32, *p* < 0.02, Wilcoxon signed rank test between the sequence time in the first 15 test trials and the last 15 training trials). Furthermore, part learners consistently suffered more shocks (made more errors) than single learners (middle panel, Fig. 1c; *T*(17) = 2.75, *p* = 0.014, paired one-sample *t*-test) in the anxiety test sessions. These results support our first behavioral hypothesis that the learning structure (part or single) modulates the DSMPA in participants.

Next, we analyzed the standard deviation (STD) of the time between button presses in a sequence across experiments (Fig. 1b, insets). We aligned the results from the STD between different presses to the “junction”, where part learners joined their pre-learnt parts of the 10-button sequence. Therefore, junction J in Fig. 1b represents the interval between buttons 4 and 5 for the participants who pre-learnt first the four-button sequence, and buttons 6 and 7 for the participants who pre-learnt first on the six button sequence. In the insets of Fig. 1b, “−1” represents a press before the junction, “+1” a press after the junction and so on. To plot the single-learner data, we assumed J to be between buttons 4 and 5 for one random half of the single learners and between buttons 6 and 7 for the other half.

We observed prominent differences in between the STD patterns of the part learners and single learners at the start of training, including a large difference at the junction (*Z* = 45, *p* = 0.045, Wilcoxon signed-rank test). This difference is not unusual considering that at the start of the 10-button sequence training, part learners probably combined their pre-learnt sequences at the junction. By the end of the training, the difference between part learners and single learners disappeared (*χ*^2^(17) = 17, *p* > 0.45, Kruskal Wallis test between the two learners across the last 15 training trials), indicating that the behaviors of the part learners and the single learners was similar by this stage. However, in the presence of anxiety, the STD difference reappeared (see inset in the yellow shaded region in Fig. 1b), but only at the junction. The bottom panel of Fig. 1c plots the absolute change of STD between the first 20 test trials and the trials from the last training session across the participants. We choose these trial numbers to correspond to the data analysis in Experiment 2 and Experiment 3 (see below). Again, the changes in part learners were observed only at the junction (*Z* = 2.07, signed rank = 38, *p* = 0.038, Wilcoxon signed rank test) (see Supplementary Fig. 1A for details).

Similar changes at the junction were observed with regard to the participant’s inter-press time and button-press error (Supplementary Fig. 1B, C), but we will focus on the inter-press STD changes henceforth, as these were the most consistent across our three experiments. The results in Fig. 1c and Supplementary Fig. 1 provide insight into the temporal dynamics of DSMPA and support our second behavioral hypothesis, that in our DMSL task, DSMPA is predominantly focused at the junction of the pre-learnt parts.

### Experiment 2

Next in Experiment 2, we analyzed the neural correlates of DSMPA using fMRI (Fig. 2) and examined our neural hypothesis that dACC activity will differentially correlate with the performance deteriorations at the junction by part- and single learners. Experiment 1 confirmed the suitability of the DMSL task for this purpose. However, the touch-screen pressing paradigm was difficult to replicate in the scanner due to constraints of space and movement artifacts. Hence, we utilized a button-pad version of the DMSL task in Experiment 2. Thirty-four new participants participated in this study, 17 as part learners and 17 as single learners. The general experiment and instructions in Experiment 2 remained the same as Experiment 1, though this time the participants pressed button sequences on a button pad with three buttons under their index, middle, and ring fingers. The screen above them (they were lying on their back inside the MRI scanner) always showed three green circles corresponding to the buttons, and the participants were asked to press the button corresponding to the circle that lit up (see Methods for details). We utilized only one button sequence in this experiment. All part learners pre-trained on the first six buttons and then the next four buttons of the sequence in separate preliminary sessions before training on the complete 10-button sequence session (see Fig. 2b). The single learners again directly trained on the 10-button sequence. Both the part- and single learners trained for the same number of trials on the complete 10-button sequence and immediately after the training took part in an anxiety test session (again with electrical shocks to induce performance anxiety) of the sequence they had learnt. We performed fMRI using the EPI sequence during the sessions (TR = 3 s, TE = 25 ms; Siemens MAGNETOM 3T Prisma. See also Methods).

Again, similar to Experiment 1, the test sessions led to an increase of the GSR in both single- (*T*(16) = 3.32, *p* = 0.004, one-sample *t*-test) and part learners (*T*(15) = 2.69, *p* = 0.01, one-sample *t*-test; one GSR value was lost). Furthermore, we also measured photo plethysmography (PPG) in the participants and introduced a questionnaire to access self-perceived cognitive anxiety in this experiment (see Methods for details). The PPG amplitudes were reduced significantly in both the single- (*Z*(16) = 2.05, *p* < 0.02, Wilcoxon signed rank test) and part learners (*Z*(16) = 2.34, *p* < 0.02, Wilcoxon signed rank test), again showing that anxiety increased in the test sessions. Similarly the scores from the questionnaire exhibited that, compared to the training session, perceived anxiety was higher in the test session in both single (across participant score: 5.1 ± 0.78 standard deviation, *T*(14) = 5.47, *p* < 0.001, one-sample *t*-test) and part learners (across participant score: 5.49 ± 0.67, *Z*(16) = 3.57, *p* < 0.001, Wilcoxon signed rank test) in Experiment 2.

Looking at the behaviors, we found consistencies with Experiment 1 in that part learners suffered more shocks (*T*(32) = 2.59, *p* = 0.014, two-sample *t*-test, top panel in Fig. 2c) than single learners. Furthermore, the change of inter-press STD was different between part learners and single learners only at the junction (*T*(32) = 2.01, *p* = 0.05, two-sample *t*-test, bottom panel of Fig. 2c). This temporal specificity allowed us to utilize an event-related design and identify the neural correlates of the DSMPA across individual trials.

We conducted a parametric event-related general linear model analysis of the anxiety test session using SPM12 (ref. ^24^). The time at which a participant pressed the seventh button in every trial was defined as the junction event. We utilized two regressors to represent the junction events: the “junction-shock” and “junction-no-shock” regressors, which were aligned respectively to the junctions of trials in which a participant did and did not suffer an electrical shock. The inter-press time between the sixth and seventh buttons from the no-shock trials (i.e., the junction) was used as a parametric regressor (only no-shock trials were considered to avoid artefacts). In addition to the junction regressors, we also included a “start-sequence” regressor, with events aligned to the presentation of the first button in every trial, a “shock” regressor, with events aligned to the shocks received by a participant, and a “success” regressor, with events aligned to the last button of each sequence trial that the participant completed successfully without receiving any shock (see Methods). In addition, we also considered the six head movement regressors in the general linear model. All task regressors (or events), except the inter-press time regressor, produced significant results only for the main effect. The inter-press time regressor yielded significant activations only for the contrast between part learners and single learners (i.e., part–single). A statistical threshold of *p* < 0.05 with family-wise-error (FWE) correction was utilized for all the analysis. We utilized a spatial threshold of five consecutive voxels (*K*_E_ = 5) unless otherwise stated.

A two-sample *t*-test of the inter-press time regressor demonstrated that, like we had expected, activity correlated with inter-press time in the dACC (peak MNI coordinates, [0, 36, 36]) and left dorsal premotor cortex (peak MNI coordinates, [−48, 8, 42]) were significantly higher in part learners than in single learners (Fig. 2d, and Supplementary Table 1). Furthermore, we found a positive correlation between dACC activity and inter-press time (*p* = 0.00017, Fig. 2e) in part learners, while interestingly, this correlation was observed to be negative in single learners (*p* = 0.0010, Fig. 2e). These observations suggest that, while a larger dACC activity results in slower inter-press time in part learners, it results in faster inter-press time in single learners. We analyzed the relationship between the differential brain activity in these areas and the number of electrical shocks and found that dACC activity was positively correlated with the number of electrical shocks in part learners (Fig. 2f, left, *R* = 0.58, *p* = 0.016), but again, negatively correlated in single learners (Fig. 2f, right, *R* = −0.64, *p* = 0.0054). By contrast, no such correlation with shocks was observed for the left premotor cortex in either part learners or single learners (*R* = 0.375, *p* = 0.14 for part learners, and *R* = 0.11, *p* = 0.67 for single learners). These findings suggest a context-dependent effect of dACC activity on motor sequence performances that caused DSMPA in part learners, but performance facilitation in single learners.

The results for all other regressors are summarized in Supplementary Table 1. Only activation in the primary visual cortex correlated with the *success* regressor. The *shock* regressor highlighted regions in the primary sensory cortex, dACC, insula, and amygdala, which are brain structures known to be involved in the processing of pain and anxiety^25^. Crucially, the dACC activity that correlated with the shock regressor (Supplementary Fig. 5) was distinct and located posterior from the dACC activity identified for DSMPA at the junctions (Fig. 2d). Both the *junction-shock* and *junction-no-shock* regressors correlated with activity in the primary motor cortex. Finally, the *start-sequence* regressor highlighted areas that have been previously implicated in sequential motor control^26^, such as the putamen, supplementary motor area, and dorsal premotor cortex.

### Experiment 3

Though we observed a strong positive correlation between dACC activation and the participant’s junction press time for part learners, the fMRI results did not clarify the reason for the activation. Specifically, it was not clear whether the dACC activation is a cause of the DSMPA in part learners, or an effect of the motor deterioration at the junction or larger number of shocks suffered. We clarified this issue in Experiment 3, in which we used repetitive TMS (rTMS) of the dACC using a double-cone TMS coil designed for deep simulation^27^. Low-frequency (i.e., 1 Hz) rTMS is known to suppress the brain activity of the target region^28^. We examined whether and how dACC activity suppression affects the DSMPA in part learners, envisioning future intervention techniques.

Experiment 3 included 31 right-handed participants. All participants were unfamiliar with TMS and experienced it for the first time (confirmed by a questionnaire). We obtained structural scans of the participants’ brains prior to the rTMS session. These scans were used to develop an inverse field map and localize the dACC (corresponding to peak MNI coordinates, [0, 36, 36], in a standardized brain, Fig. 2d) in each participant’s brain (see Methods for details).

All participants in Experiment 3 participated as part learners and performed a similar DMSL task on a button pad as in Experiment 2, albeit in a sitting posture (Fig. 3a; see Methods for details). All participants pre-trained on the first six buttons and then the next four buttons of the sequence in separate preliminary sessions before training on the complete 10-button sequence session (Fig. 3b). Following the training, half of the participants underwent rTMS to the dACC (1 Hz, 320 pulses; these participants are referred to as *TMS part learners*). The remaining participants (*SHAM part learners*) were subjected to a sham stimulation (1 Hz, 320 pulses) to the dACC with a 1-cm thick plastic board placed between the scalp and the TMS coil. Both groups took part in an anxiety test session immediately after the (TMS/SHAM) stimulation. The test sessions led to an increase of the GSR in TMS part learners (*T*(14) = 4.95, *p* < 0.001, one-sample *t*-test) and SHAM part learners (*T*(13) = 4.4, *p* < 0.001, one-sample *t*-test), but these GSR changes were not different between the two groups (*T*(27) < 0.65, *p* > 0.52, two-sample *t*-test). The PPG values also showed the increase in anxiety test session in TMS part learners (*T*(14) = 2.21, *p* = 0.04, one-sample *t*-test) and SHAM part learners (*T*(13) = 2.29, *p* < 0.04, one-sample *t*-test). Furthermore, similar to Experiment 2, the scores from the questionnaire indicated that, compared to the training session, perceived anxiety was higher in the test session in both TMS part learners (across participant score: 4.65 ± 1.65, *T*(14) = 2.36, *p* < 0.035, one-sample *t*-test) and SHAM part learners (across participant score: 4.61 ± 1.05, *T*(13) = 2.17, *p* < 0.05, one-sample *t*-test) in Experiment 3. These changes were however similar between the TMS and SHAM part learners (*p* = 0.25 for PPG change, two-sample *t*-test; *p* = 0.89 for the questionnaire scores, Wilcoxon rank-sum test). These results suggest that the TMS did not change the level of anxiety experienced by the participants in the test session.

As for behaviors, no differences were observed between press behaviors by the TMS part learners and SHAM part learners (Supplementary Fig. 3) during training. A two-way ANOVA showed that their press sequence time through the training sessions were the same (no main effect between groups (*F*(1, 1080) = 1.82, *p* = 0.18), and there was no interaction between groups and trials across the last training session (*F*(39, 1080) = 0.22, *p* > 0.90)). Similarly, the inter-press STD patterns at the end of the training sessions were similar (Supplementary Fig. 3).

However, there were stark differences in the anxiety test session after the rTMS. First, the number of electrical shocks suffered by TMS part learners was significantly less than SHAM part learners (*Z* = 2.08, rank sum = 177.5, *p* = 0.037, Wilcoxon rank-sum test) (see top panel Fig. 3c). Second, the change in STD across the junction was different between the two groups (Fig. 3c, bottom panel). While SHAM part learners displayed a peak at the junction similar to part learners in Experiments 1 and 2 (compare red traces in Figs 1c, 2c and 3c), this peak disappeared in TMS part learners. Instead, the STD pattern of TMS part learners was similar to single learners in Experiment 2 (*Z* = 0.72, rank sum = 261, *p* > 0.47, Wilcoxon rank-sum test). These results show that rTMS of the dACC changed the behavior of TMS part learners, improving their behavior in the presence of anxiety to the level of single learners (compare Figs. 2c and 3c).

## Discussion

The present study introduced a novel DMSL task to reliably measure and quantify the DSMPA. Using this task, we demonstrated that part learners were prone to DSMPA predominantly at the junctions of pre-learnt motor sequences (Experiment 1). Next, an fMRI version of the DMSL task (Experiment 2) showed that dACC activity at the junctions was positively correlated with both inter-press time and the number of electrical shocks in part learners, while this correlation was negative for single learners. Finally, to evaluate the role of the dACC in DSMPA, we conducted 1-Hz repetitive TMS of the dACC prior to the DMSL task. TMS of the dACC attenuated the part-learner’s DSMPA at junctions (Experiment 3), demonstrating a causal relationship between dACC activity and DSMPA.

Behavioral results throughout the three experiments demonstrated that different training strategies (i.e., learning a sequence of action in parts, or as a whole) can affect sequential motor control under anxiety (i.e., DSMPA) differently. Our experiments provided the first insights into the temporal dynamics of anxiety-induced motor deteriorations showing that DSMPA is local in time and space at the junctions. This property of DSMPA in the DSML task enabled us to adopt an event-related design for fMRI analysis and to identify neural correlates of DSMPA.

Utilizing fMRI, we observed that part learners exhibited a positive correlation between the dACC activity at the junctions and inter-press time, and between the dACC activity at the junctions and the number of shocks. On the other hand, interestingly, the single learners showed a negative correlation for both. This context-dependent activation of the dACC under anxiety is similar to observations by recent studies that suggest dACC activity represents explorations of alternative actions (through positive modulations), as well as commitment to a selected action (through negative modulations)^15,20,21^. Our data thus suggest that under the influence of anxiety, part learners may engage the dACC more to consider action alternatives at the junctions in the presence of anxiety, leading to a delay in the response. On the other hand, because single learners have learnt the sequence in only one way (as a whole), the negative correlation of the dACC activation at the junction represents the tendency of single learners to persist with the learnt actions. In other words, DSMPA in part learners is likely to be driven by the erroneous cognitive control of motor sequences due to anxiety. Crucially, the dACC activity that correlated with DSMPA (Fig. 2d) was distinct from the shock-dependent dACC activity (Supplementary Table 1, and Supplementary Fig. 5), indicating that the dACC activity causing DSMPA is independent of the processing or imagination of shocks per se.

This view that DSMPA is driven by erroneous cognitive control is closely related to the self-focus theories of performance anxiety^1–3^, in which performance deterioration is assumed to be caused by the re-activation of explicit monitoring processes due to anxiety. Further, this mechanism of DSMPA is distinct from the mechanisms of risk aversion and motivation that have been implicated in the anxiety-induced effects on individual actions^6–10^, suggesting that motor performance deterioration in the real world is caused by at least two mechanisms: the effects of anxiety in sequential actions demonstrated in the present study and the anxiety effect previously observed in individual actions^6–10^.

Brain activity occurring at the end of the sequences may represent positive and negative feedback or a prediction error^13,14^. We found that activity in the anterior caudate nucleus was activated due to shocks (Supplementary Fig. 6, although this information was not included in Supplementary Table 1 because *K*_E_ = 4), while only the primary visual cortex was activated after successful trials. Therefore, at present evidence linking DSMPA and hierarchical reinforcement learning is partial, and further research is needed to address this issue.

Although double-cone TMS has been successfully applied to dACC in previous studies^27,29^, it is technically difficult to rule out the possibility that rTMS to the dACC also modulated activity in the pre-supplementary motor area (Pre-SMA), which has also been implicated in sequential motor performance and learning^26^. Previous studies have however reported that the TMS stimulation of Pre-SMA induced task switches^30,31^ and delayed the start of the motor sequence^31^. In the present study, we did not observe these changes, suggesting that the present results are mostly attributable to stimulation of the dACC.

Finally, another interesting question is whether rTMS of the dACC reduced the perception of anxiety or the sensitivity of motor apparatus to the anxiety. We recorded GSR in all experiments, and PPG and cognitive anxiety scores in Experiments 2 and 3, but did not find any significant difference in any of these measures between part learners and single learners. This observation, together with our fMRI finding that, in addition to the dACC the dorsal premotor cortex is correlated with the inter-press time in part learners, supports the reduction of sensitivity of the motor apparatus to anxiety (associated with the selection of the motor sequences), but further studies are needed to clarify this point. However, irrespective of the two possibilities, our findings that DSMPA can be attenuated using training strategies and TMS should be interesting for cognitive and sports scientists, and may yield new techniques for interventions against DSMPA in sports and music scenarios, where sequence learning and sequence performance are common place.

## Methods

### Participants

Eighty-two Participants (33 females) participated in the three experiments: 18 in Experiment 1, 34 in Experiment 2, and 30 in Experiment 3. The participants gave informed consent for participating in the experiments, and the experiments were approved by the local ethics committee at the Centre for Information and Neural Networks (CINET), Osaka, Japan.

### Experiment 1: participants and paradigm

Experiment 1 included 18 right-handed participants. The participants sat on a chair in front of a touch screen (ET1915L-8CJA-1-BG-G, elo TOUCH SOLUTIONS Inc.). They were presented with a sequence of green circles across the screen as “buttons” one at a time, which they pressed/touched with the index finger of their right hand. Each correct press extinguished the touched button and presented the next button in a sequence. The participants worked in repeated training trials of each sequence, first in training sessions in which they learnt the sequence. Then they completed the anxiety test session, in which they performed the learnt sequence and were punished with an electrical shock using the BIOPAC system (BIOPAC Systems, Inc.) if they missed a button or were “too slow” (see later for details) in their presses.

The experiment started with instructions that briefly explained the task to the participants. They were told that the experiment would be used to “understand the effects of anxiety on motor performance” without any specifics. A demonstration of the task was performed by the experimenter to help participants visualize the task. The button sequence shown in the demonstration was different from those used in the experiments. We then calibrated the electrical stimulator for the participants as follows.

We fixed the electrical stimulator electrodes on the left forearm of the participants. We utilized a short Gaussian burst of an electrical current lasting 25 ms as a shock. We asked the participants to close their eyes, and starting from zero, we then slowly increased the amplitude of the shock until the participants reported perceiving it on their skin. Following this, the participants were instructed that the shock will be slowly increased in steps and that they should report the *threshold shock level* at which they think they would feel anxious during performance. To help them converge on this threshold, they were instructed to think of the level at which they may not mind to get the shock once, but would be upset if subjected to three times in immediate repetition. The participants were encouraged to try higher levels of shock and then return back to a lower level if they felt uncomfortable. Overall, this procedure was designed to reduce the surprise the participants felt on their first shock (as we observed that a surprise would make them report a lower threshold shock level), and encourage them to truly find a level they would be anxious about.

Following the stimulator calibration, GSR sensor electrodes were affixed on the participants’ right index and middle fingers (BIOPAC Systems, Inc.). The participants were then allowed to try the demonstration sequence of presses three times before moving on to the experiment.

### Experiment 1: sessions

We utilized two distinct spatial button sequences in Experiment 1, and every participant trained on both of them as a single learner in one sequence and as a part learner in the other. Overall, each participant took part in 10 experiment sessions, each with 40 trials. The order of the single/part learning sessions and the sequence utilized for each were balanced across participants. As part learners, participants pre-learnt parts of a 10-button sequence. One half of them trained on the first six buttons and the next four buttons of the sequence in separate preliminary sessions (two alternating 40 trial sessions for each training sequence), and the other half trained on the first four buttons and the next six buttons of the sequence in separate preliminary sessions. All participants then went on to train on to the complete 10-button sequence session (see paradigm description in Fig. 1a). As single learners, participants directly trained on the 10-button sequence. For each sequence, participants were instructed to “focus your training on speeding up your press sequence as much as possible and make sure you don’t miss the buttons”. Furthermore, before moving on to the 10-button training, part learners were instructed that the “10-button sequence is an assimilation of the previously learnt part sequences, such that the 6 (or 4)-button sequence you learnt first would be presented again and would be immediately followed by the 4 (or 6)-button sequence you learnt after. Again, please focus your training on speeding up your press sequence as much as possible and make sure you don’t miss the buttons”.

After each trial of presses, the participants had a rest of 3 s (Fig. 1a) before moving onto the next press trial. Both single- and part learners were trained for the same number of trials on the complete 10-button sequence and immediately after this training took part in an anxiety test session of the 10-button sequence they had learnt. Before this session, the participants were instructed that “you will suffer an electric shock if you miss any button. You are allowed to slow your presses, but if it becomes too slow and below a “time threshold”, you will suffer a shock at the end of the trial”. Unknown to the participants, the “time threshold” was tuned for each participant and set to 1.5 times their average sequence time in their last ten training trials.

### Experiment 1: behavioral data analysis

We analyzed the mean and STD of the changes in the total sequence press time in each trial, the time between individual button presses (inter-press time) in a sequence, and the spatial error of the presses, which were measured as the distance of the presses from the center of the buttons. We focused on the measures in the last two (ten button) training sessions and the last anxiety test session when comparing the behaviors of single- and part learners.

### Experiment 1: GSR analysis

To assess the increase of anxiety in the participants in the test session, we assessed the GSR level at the start of the test session. To avoid any noise in the GSR due to the shocks, for each participant, we considered the average GSR from the start of the experiment, over the trials before the first shock (until a maximum of five trials). This value from a participant was compared with the average GSR from the first five trials of the last training session by the same participant. We compared between GSR’s from the start of the sessions to equalize any anxiety due to the start of the task. This difference was averaged across participants to assess the increase of anxiety in the test session.

### Experiment 2: participants and sessions

Experiment 2 involved 34 right-handed participants. Seventeen participants participated as single learners, while the other 17 participated as part learners. The participants worked on a motor sequence learning task similar to Experiment 1. The participants lay on their back on the MRI scanner bed and pressed a 3-button keypad with their right index, middle, and ring fingers. A screen above them (they were lying inside the MRI scanner) showed three green circles corresponding to the buttons, and the participants were asked to press the button corresponding to the circle that lights up. We utilized only one button sequence in this experiment. All part learners were pre-trained on the first six buttons and then the next four buttons of the sequence in separate preliminary sessions before training on the complete 10-button sequence session (Fig. 2b). Single learners were directly trained on the 10-button sequence. Both single- and part learners trained for the same number of trials on the complete 10-button sequence and immediately after, took part in the anxiety test session (again with electrical shocks to induce performance anxiety) of the 10-button sequence they had learnt.

All participants took part in a short initialization (outside the scanner) before the fMRI experiment. The initialization was done between 1 and 3 days before the fMRI experiment. In the initialization, the participants were instructed on the experiment task and purpose similar to Experiment 1. They were shown a demonstration of the button-press task on a scanner mockup with a bed and a visual feedback screen fixed above. Following this, the part learners were asked to undergo four training sessions (20 trials each), alternating between the 6-button/4-button sequences twice. Single learners were allowed to try a demonstrated sequence different from the one in the experiment for five trials, but did not undergo any other training.

Finally, because most participants were to experience electrical shocks for the first time, all were given a demonstration of the shocks by the electrical stimulator. The shock electrodes were affixed on their left forearm, and the shock magnitude was slowly increased from zero until they could feel the stimulation. The stimulations were not increased above this level during the initialization.

### Experiment 2: fMRI experiment steps

The participants started the fMRI experiment by filling the relevant ethics and procedural forms. The GSR electrodes were then affixed on their left index and middle fingers. PPG was collected from their left little finger. The electrical stimulator electrodes were affixed onto their left forearm. Following this, we calibrated the electrical stimulator using the same procedure as in Experiment 1. The participants then started the experiment after the fMRI scanner initialization.

The part learners performed five sessions inside the scanner (Fig. 2b): one 20 trial session of the 6-button sequence and one 20 trial session of the 4-button sequence, followed by two 20 trial sessions of the 10-button sequence. Finally, they conducted the 10-button anxiety test session. Single learners performed only three sessions (Fig. 2b): two 20 trial sessions of the 10-button sequence, followed by the 10-button anxiety test session.

The instructions given to the participants were the same as in Experiment 1. Both the part learners and single learners were asked to “focus your training on speeding up your press sequence as much as possible and make sure you don’t miss the buttons”. Furthermore, like in Experiment 1, before moving on to the 10-button training, the part learners were instructed that the “10-button sequence is an assimilation of the previously learnt part sequences, such that the 6-button sequence you leant first would be presented again and would be immediately followed by the 4-button sequence you learnt after. Again, please focus your training on speeding up your press sequence as much as possible and make sure you don’t miss the buttons”. Before the test sessions, both the part learners and single learners were told that “you will suffer an electric shock if you miss any button. You are allowed to slow your presses, but if it becomes too slow and below a ‘time threshold’, you will suffer a shock at the end of the trial”. Unknown to the participants, the “time threshold” was tuned for each participant and set to 1.5 times their average sequence time in their last ten training trials similar to Experiment 1.

### Experiment 2: fMRI image acquisition

MRI scanning was performed on a Siemens 3T Prisma scanner at the CiNET using an EPI sequence with the following parameters: repetition time (TR) = 3000 ms, echo time (TE) = 25 ms, flip angle = 90°, matrix = 64 × 64, field of view (FOV) = 192 mm, slice thickness = 3 mm, gap = 0 mm, ascending interleaved slice acquisition of 51 axial slices. High-resolution T1-weighted anatomical scans were acquired using an MPRAGE pulse sequence (TR = 2000 ms, TE = 1.98 ms, FOV = 256 mm, image matrix 256 × 256, slice thickness = 1 mm).

### Experiment 2: behavioral data analysis

After observing the results in Experiment 1, we concentrated our analysis to the mean and STD of the changes in the total sequence press time in each trial and the STD of time between individual button presses (inter-press time STD).

Corresponding to the fMRI analysis, the change in the inter-press time STD was calculated between the entire test sessions compared to the entire last training session.

### Experiment 2: GSR and PPG analysis

To assess the increase of anxiety in the participants in the test session, we assessed the GSR and PPG levels at the start of the test session. Like in Experiment 1, we considered the average GSR and PPG from the start of the experiment, until the trials before the first shock (until a maximum of five trials). For PPG, we considered the average amplitude of the pulses as a measure of anxiety. Note that the amplitude of PPG decreases with an increase of anxiety.

The values of GSR and PPG from a participant were compared with the GSR and PPG from the first five trials of the last training session by the same participant. This difference was averaged across participants to assess the increase of anxiety in the test session.

### Experiment 2: Self-perceived Anxiety Questionnaire

After the end of the fMRI experiment, we asked participants to fill a questionnaire to assess their self-perceived level of anxiety. Because we are interested in performance anxiety, we found that state anxiety scales that deal with chronic anxiety (like the Speilberger scale^32^) to be unsuitable for our purpose. We are more interested in a scale similar to the performance anxiety scales proposed in literature (like Smith et al^33^; Çirakoğlu and Şentürk^34^) but these are designed more to check anxiety related to social worries related to performance (e.g., fear of being seen as untalented), which are not directly relevant to our task. We therefore omitted the social question and selected questions related to physical characteristics from Smith et al.^33^, which related to attention, muscle stiffness, etc., and added three questions of our own to check fear, anxiety (we could do this because Japanese has a specific term, “kincho”, which best translates to “anxiety/worry/fear”, but is understood clearly by Japanese as the anxiety related to performance) and temporal perception.

Our questionnaire required participants to answer nine questions/statements, which they were asked to score on a Likert scale of 1 to 7. The participants were instructed to give a score of 1 if they “disagreed strongly with the statement”, 4 if they “agreed”, and 7 if they strongly agreed. The scores from the first seven statements were averaged and checked for being above a value of 4 to check whether the anxiety test sessions increased their level of anxiety. The last two statements were used to assess possible frustration in the task.

The statements presented were as follows

S1. I was anxious during the test session.

S2. Compared to the training session, my button-press behavior was different in the test session.

S3. Compared to the training session, I was anxious during the test session.

S4. Compared to the training session, I was more afraid in the test session.

S5. Compared to the training session, I was faster or slower in my button presses during the test session.

S6. Compared to the training session, my hand was stiffer during the test session.

S7. Compared to the training session, my attention level was higher during the test session.

S8. Compared to the training session, I was more irritated during the test session.

S9. Compared to the training session, the test sessions felt longer.

### Experiment 2: fMRI data analysis

We used SPM12 (http://www.fil.ion.ucl.ac.uk/spm) for the MRI data preprocessing and analysis. Preprocessing included motion correction, co-registering to the participant’s anatomical image, and spatial normalization to the standard Montreal Neurological Institute (MNI) template with a resampled voxel size of 2 mm. Co-registered EPIs were normalized using an anatomical normalization parameter. Spatial smoothing was done using a 6-mm Gaussian kernel. Serial autocorrelation was modeled as a first-order autoregressive model, and the data were high-pass filtered at a cutoff of 128 s.

### Experiment 2: general analysis methods

Because we wished to identify the neural basis of the change in the inter-press time at the junction in the test session, we performed a general linear model (GLM) analyses on the functional data. For first-level GLM analysis, we included two event regressors representing the times at which each subject passed the *junction*; i.e., when he/she pressed the seventh button in the sequence (this is because in this experiment, all part learners pre-learnt the first six buttons and then the next four buttons). We separated the junction event into the “junction-shock” and “junction-no-shock” events to represent the junctions in trials in which the participant suffered an electrical shock or not, respectively, and used them as regressors. The inter-press time in no-shock trials between the sixth and seventh buttons (i.e., the junction) was used as a parametric regressor (we only considered no-shock trials to avoid artefacts). The same procedure was adopted for single learners even though the junction did not signify any event for them. In addition to these junction regressors, we also included a “start-sequence” regressor, with events aligned to the presentation of the first button in every trial, a “shock” regressor, with events aligned to the shocks received by participant, and a “success” regressor, with events aligned to the last button of each sequence trial which the participant completed successfully without receiving any shock. Including the above six task regressors and six movement regressors, our general linear model consisted of 12 regressors. All events were modeled with a duration of 0 s. The individual contrast images were then processed in a second-level random-effects analysis.

Specifically, these regressors produced significant results only for their main effect (one-sample *t*-test) except the inter-press time, which yielded significant results for the contrast between part learners and single learners (i.e., part–single, two-sample *t*-test). To correct for multiple comparisons, we utilized the family-wise error (FWE) correction across the whole brain at *P* < 0.05, with a spatial threshold of five consecutive voxels (i.e., *K*_E_ = 5).

Finally, to examine the relationship between the contrast of the brain activity between part learners and single learners, and the number of electrical shocks actually received, we plotted the participant’s beta values for the inter-press time regressor and the number of shocks and conducted a linear regression analysis on this data (see Fig. 2f).

### Experiment 3: participants and sessions

Experiment 3 involved 30 right-handed participants. All participants participated as part learners. One participant who was very slow in the task was excluded from the analysis. The motor sequence learning task and feedback were the same as in Experiment 2. However, in Experiment 3, the button presses were performed in a sitting position (see Fig. 3a) in order for us to use transcranial magnetic stimulation (TMS) between sessions.

All participants had a structural MRI session before the TMS experiment, in which we obtained T1 structural images. This image was used to localize the dorsal ACC (as explained below) for TMS. The participants did not undergo any other initialization before the TMS experiment day.

The participants started the TMS experiment by filling the relevant ethics and procedural forms. The GSR electrodes were then affixed on their left index and middle fingers. PPG was collected from their left little finger. The measurements were conducted using the BIOPAC system.

The participants started with four initial sessions similar to the initialization of Experiment 2. They performed two alternating sessions, each of 20 trials of the 6-button sequence and 20 trials of the 4-button sequence, similar to the initialization of Experiment 2. This was followed by a 20 min break during which time the electrical stimulator electrodes were affixed onto the participant’s left forearm and calibrated, similar to Experiments 1 and 2. This was followed by the TMS setup.

### Experiment 3: TMS setup and localization

A customized double-cone coil was attached to a Magstim Rapid (Magstim). We selected this device because it has been used in previous studies that stimulated the dACC^27,35^. For the location of the dACC, we chose the MNI coordinates (0, 36, 36), where we identified the maximum differential activity in the correlation with the inter-press time (Fig. 2d) between part learners and single learners. The coordinates were individually adjusted using the inverse normalization method embedded in SPM12 (normalization based on the inverse deformation field). We also individually localized the toe area in the primary motor cortex as the MNI coordinates (−8, −22, 64) following previous studies^36,37^. Brainsight (Rogue Research Inc.) was used to register the stimulus sites onto the individual T1 image. The Brainsight navigation procedure allowed for precise placement of the coil on the distal dACC and the reference toe area.

### Experiment 3: TMS intensity calibration

To calibrate the TMS intensity for an individual, we first applied the TMS on the toe area in the primary motor cortex and gradually increased the stimulus intensity until we visibly observed a toe movement (which we call the motor threshold). The motor threshold value was used to calibrate the TMS on the dACC. We utilized a TMS intensity corresponding to 110% of the motor threshold on the ACC following previous studies^27,35^. A total of 320 single pulses were given to the dACC repetitively at a frequency of 1 Hz. Off-line low-frequency stimulation is known to have an inhibitory after-effect on the stimulated brain areas^38^. Because the task duration was less than 2.5 min, a 5-min stimulation was sufficient to produce suppressive effects through our experiments on the dACC^28^.

### Experiment 3: experiment sessions

Following the TMS setup, the participants did two 40 trial sessions of the 6-button sequence, and two 40 trial sessions of the 4-button sequence, and two 20 trial sessions of the 10-button sequence. This was followed by a stimulation period in which 15 participants were subjected to 320 pulses of the 1-Hz TMS. The remaining 14 participants were subjected to a SHAM stimulation, where a 1-cm thick plastic board was put between the scalp and the TMS coil. Therefore, the participants sensed the vibration of the coil, but the stimulation did not affect their brain activity. Importantly, none of the 29 participants had prior experience with TMS.

Finally, after the stimulation, all participants went on to the 10-button anxiety test session. All instructions for the session in Experiment 3 were the same as in Experiment 2.

### Experiment 3: behavioral data analysis

All data are plotted in Fig. 3 and Supplementary Fig. 3 with participants grouped as TMS and SHAM part learners.

### Experiment 3: GSR, PPG, Questionnaire

GSR and PPG were collected and analyzed as in Experiment 2. All participants filled the Self-perceived Anxiety Questionnaire as in Experiment 2.

### Statistical comparisons

While performing comparisons for all experiments, we checked for the normality of the datasets using the Shapiro–Wilk test. Parametric analysis (*t*-test or ANOVA) was used for comparisons when the data groups were normal, and non-parametric tests (Wilcoxon signed rank or rank sum, or Kruskal Wallis test) were used when one or more of the datasets were non-normal. All statistical tests were two-tailed in nature.

### Reporting summary

Further information on research design is available in the Nature Research Reporting Summary linked to this article.

## Supplementary information

Supplementary Information

Reporting Summary

## Data Availability

The data that support the findings of this study are available from the corresponding authors upon reasonable request.

## Code availability

All data analysis codes will be made available by the corresponding authors on request.
