# Supplementary material for: Activity in the dorsal ACC causes deterioration of sequential motor performance due to anxiety
Source: Nat Commun. 2019 Sep 19;10:4287. doi: 10.1038/s41467-019-12205-6 (PMC6753143; doi:10.1038/s41467-019-12205-6)
Supplement: Supplementary file 1 — Supplementary Information [file 41467_2019_12205_MOESM1_ESM.pdf]

Supplementary material for-

Activity in the dorsal ACC causes deterioration of sequential motor performance due to anxiety

Gowrishankar Ganesh, Takehiro Minamoto, and Masahiko Haruno

Supplementary Table 1

	Area	*Peak MNI*			K_E	*T*
						Inter-press time at junction (part-single)
	dACC	0	36	36	7	5.29
	L PMd	-48	8	42	10	5.33
						success (part+single)
	V1	-2	-78	0	156	5.91
						shock (part+single) (P<0.001, uncorrected)
	R Primary Sensory	-50	-30	32	347	5.23
	L Primary Sensory	-56	-20	26	34	4.28
	dACC	-8	16	36	22	4.60
	R PMd	48	20	28	124	4.69
	R Insula	32	14	-8	30	4.32
	R STS	68	-38	12	313	5.15
	L STS	-62	-48	8	86	4.37
	L Amygdala	-28	2	-18	37	4.62
						Junction-no-shock (part+single)
	R STS	60	-16	22	34	7.24
	L M1	-40	-18	36	11	6.23
	L STS	-56	-18	20	87	7.93
	SMA	4	8	52	42	7.09
	R PMd	38	-4	50	17	7.46
	L Cerebellum	-34	-62	-20	39	7.96
						Junction-shock (part+single)
	L M1	-50	-28	48	64	7.64
	R M1	44	-42	52	6	6.57
	R TPJ	54	-44	40	77	8.37
	L Amygdala	-24	-4	-12	5	6.71
						Start-sequence (part+single)
	L Thalamus	-6	-26	-2	410	12.72
	R Thalamus	8	-22	0	311	12.92
	L Putamen	-28	2	0	1331	12.59
	R Putamen	24	8	2	974	12.20
	L M1	-30	-26	50	1012	12.09
	SMA	-6	0	58	517	11.03
	L Cerebellum	-24	-52	-24	326	11.29
	R Cerebellum	20	-54	-20	1219	12.36
	R STS	38	-32	18	24	7.57
	L STS	-46	-26	20	330	9.39
	L V1	-14	-88	-10	240	8.85
	R PMd	40	-14	54	133	9.27

Supplementary Table 1: Results of the GLM analysis of the fMRI data. Except for the inter-press time at the junction, no other regressors produced significant activity for the subtraction between part-learners and single-learners. The statistical threshold was P< 0.05, FWE corrected, with a spatial threshold of 5 consecutive voxels (K_E). We used P<0.001, uncorrected for multiple comparisons, to show the locations of activity correlated with the shock regresssor. L: left, R: right, dACC: dorsal anterior cingulate cortex, PMd: dorsal premotor cortex, STS: superior temporal sulcus, SMA: supplementary motor area, M1: primary motor cortex, TPJ: temproparietal junction.

Supplementary figures

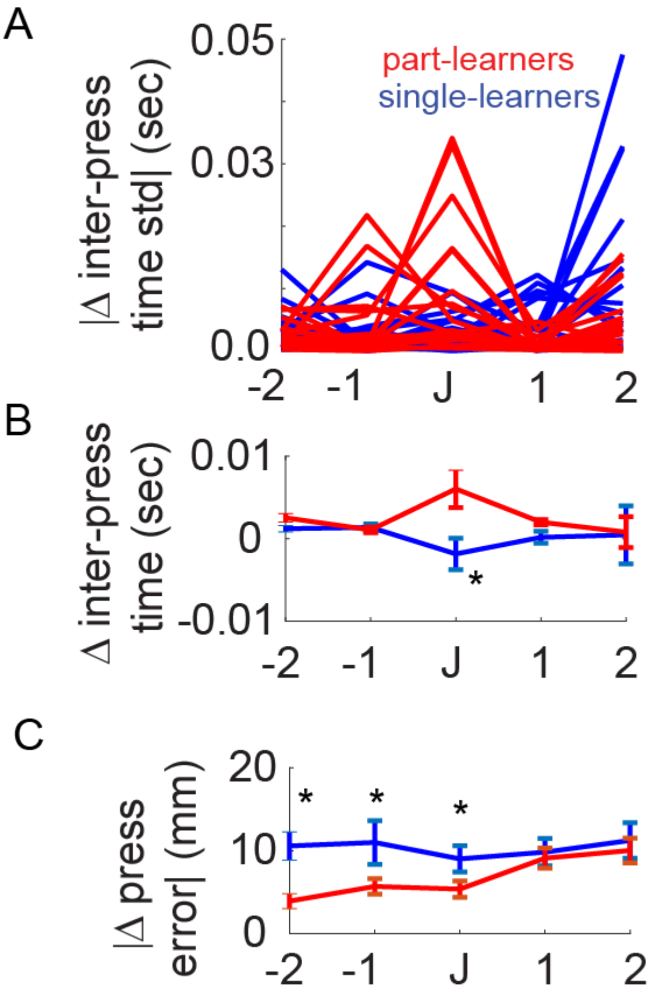

Supplementary Fig. 1: A) Individual changes in inter-press time standard deviation (std) when participants participated as part-learners (red traces) or single-learners (blue traces) in Experiment 1. These plots were averaged to plot the bottom panel of Fig. 1C. The across participant change in inter-press time (B) and spatial press error (C) when the participants started the anxiety test session. Note the importance of the junction, J, in the behaviors. The data plots in (B) and (C) show a significant difference between the part-learners and single-leaners ($p<0.003$ and $p<0.01$ respectively, main effect of learning type in 2-way ANOVA). Asterisks show significant difference ($p<0.05$, one sample t-test) between the learners at a particular inter-button interval.

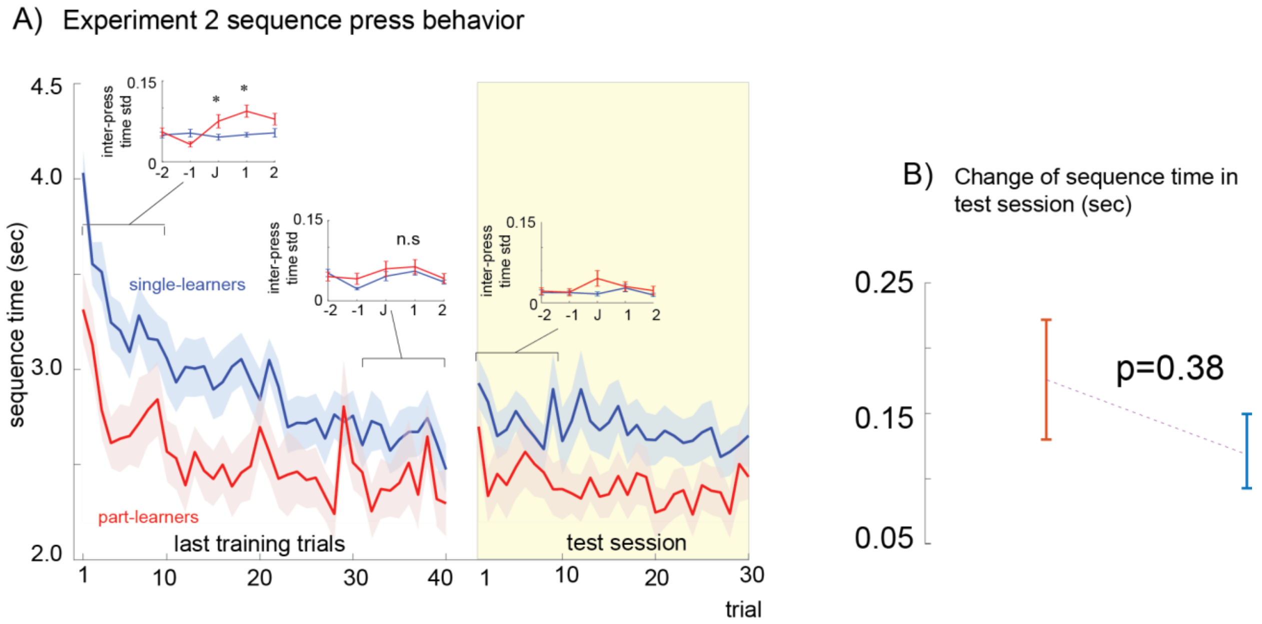

Supplementary Fig. 2: A) Learning curves in Experiment 2. Inset figures plot the local variance in inter-press time std in the initial and last trials. Asterisks shows significant difference ($p<0.05$, 2 sample t-test) between the single and part learners. B) The across subject change in sequence time in the test session indicated a larger change in part-learners than in single-learners, similar to Experiment 1, but the difference did not reach significance ($p=0.38$, Wilcoxon ranksum test). Error bars represent standard error.

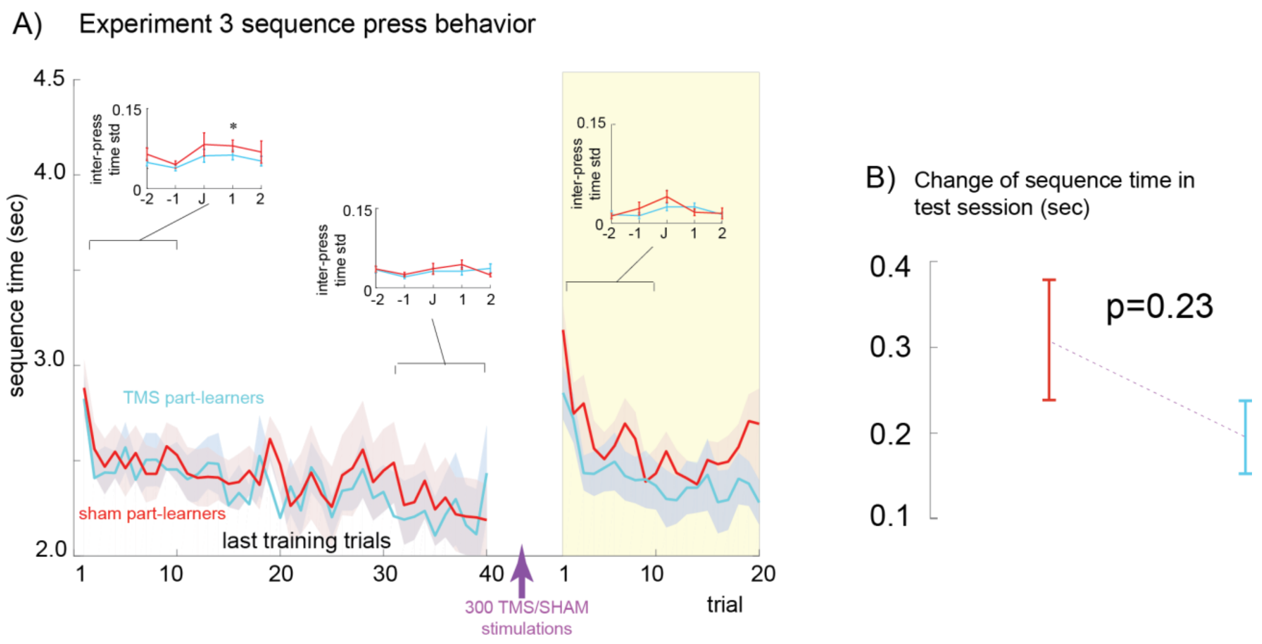

Supplementary Fig. 3: A) Learning curves in Experiment 3. Inset figures plot the local variance in inter-press time std in the initial and last trials. Asterisks shows significant difference (p<0.05, 2 sample t-test) between the single and part learners. B) The across subject change in sequence time in the test session indicated a larger change in part-learners than in single-learners, but the difference did not reach significance (p=0.23, Wicoxon ranksum test).

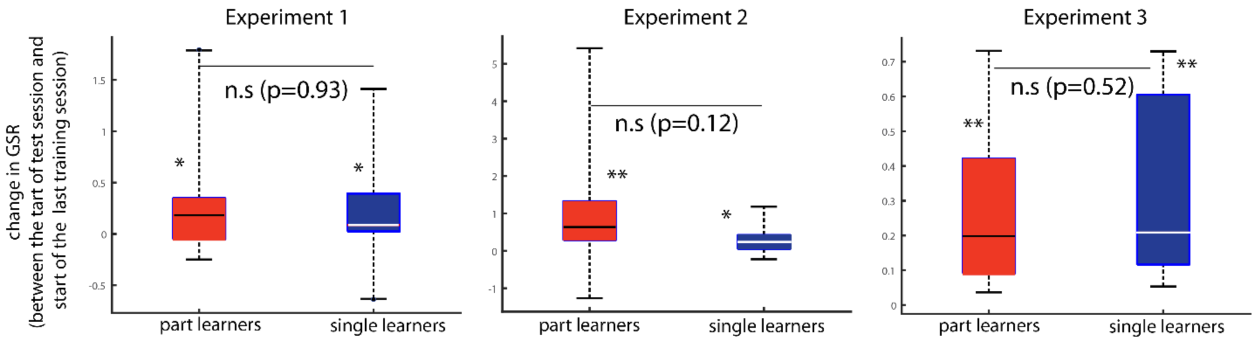

Supplementary Fig. 4: Change of GSR: Difference in average GSR values from the start of the test session (until the trial in which they received the first shock or trial number 5, whichever was less) and the first 5 trials of the last training session averaged across single-learners (blue bars) and part-learners (red bars) in our three experiments. The rise in GSR was significant for all participants in every experiment, demonstrating that the test session consistently increased GSR. The increase was similar between single- and parts-learners. P values on individual GSR changes were calculated using one sample t-tests. Comparisons between bars were done using 2-sample t-tests.

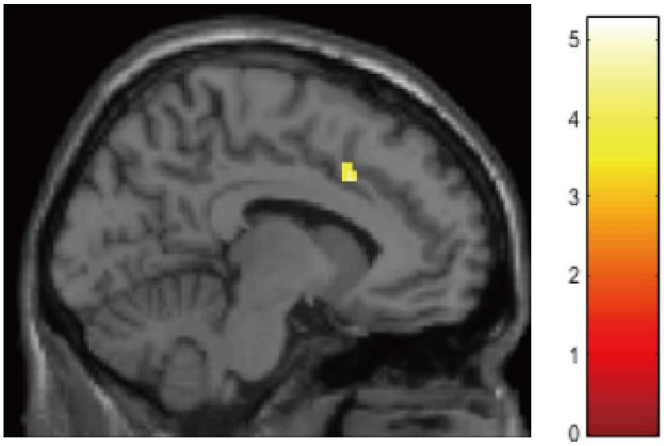

Supplementary Fig. 5: dACC activity isolated by the shock regressor (shown with p<0.001 for display purposes). Activity correlated with electrical shocks was identified in the dorsal ACC, whose peak voxel was [-8, 16, 36] in MNI coordinates. The main anxiety-related dorsal ACC activity (Fig. 2D, its peak MNI coordinates were [0, 36, 36]) was located more anterior and did not overlap with the shock-related activity. Color bar codes the T values.

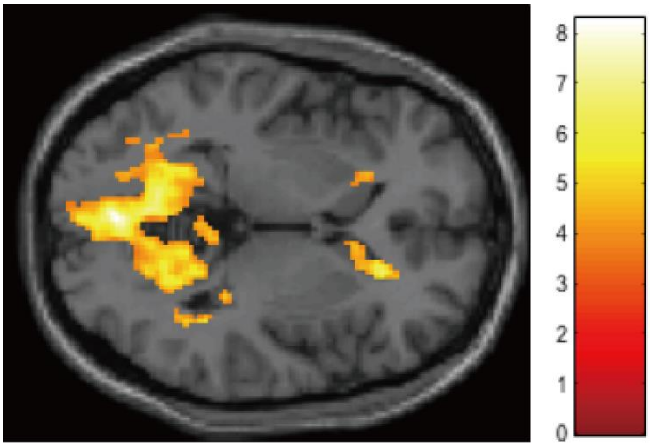

Supplementary Fig. 6: Anterior caudate activity correlated with the success regressor: While only some activation in the V1 was observed for K_E=5, setting K_E=4 highlighted activity that correlated with the success regressor in the bilateral anterior striatum, with the peak voxel at [20, 30, 0] in MNI coordinates (p<0.05 FWE corrected, 4 consecutive voxels (K_E), shown here with p<0.001 for display purposes). Color bar codes the T values.
