# Supplementary material for: Activity in the dorsal ACC causes deterioration of sequential motor performance due to anxiety
Source: Nat Commun. 2019 Sep 19;10:4287. doi: 10.1038/s41467-019-12205-6 (PMC6753143; doi:10.1038/s41467-019-12205-6)
Supplement: Supplementary file 2 — Reporting Summary [file 41467_2019_12205_MOESM2_ESM.pdf]

nature research

Corresponding author(s): Gowrishankar Ganesh
Masahiko Haruno

Last updated by author(s): Aug 8, 2019

Reporting Summary

Nature Research wishes to improve the reproducibility of the work that we publish. This form provides structure for consistency and transparency in reporting. For further information on Nature Research policies, see Authors & Referees and the Editorial Policy Checklist .

Statistics

For all statistical analyses, confirm that the following items are present in the figure legend, table legend, main text, or Methods section.

n/a	Confirmed	
☐	☒	The exact sample size (*n*) for each experimental group/condition, given as a discrete number and unit of measurement
☐	☒	A statement on whether measurements were taken from distinct samples or whether the same sample was measured repeatedly
☐	☒	The statistical test(s) used AND whether they are one- or two-sided<br>*Only common tests should be described solely by name; describe more complex techniques in the Methods section.*
☐	☒	A description of all covariates tested
☐	☒	A description of any assumptions or corrections, such as tests of normality and adjustment for multiple comparisons
☐	☒	A full description of the statistical parameters including central tendency (e.g. means) or other basic estimates (e.g. regression coefficient) AND variation (e.g. standard deviation) or associated estimates of uncertainty (e.g. confidence intervals)
☐	☒	For null hypothesis testing, the test statistic (e.g. *F*, *t*, *r*) with confidence intervals, effect sizes, degrees of freedom and *P* value noted<br>*Give P values as exact values whenever suitable.*
☒	☐	For Bayesian analysis, information on the choice of priors and Markov chain Monte Carlo settings
☒	☐	For hierarchical and complex designs, identification of the appropriate level for tests and full reporting of outcomes
☐	☒	Estimates of effect sizes (e.g. Cohen's *d*, Pearson's *r*), indicating how they were calculated

Our web collection on statistics for biologists contains articles on many of the points above.

Software and code

Policy information about availability of computer code

Data collection	matlab, Brainsight
Data analysis	matlab, SPM

For manuscripts utilizing custom algorithms or software that are central to the research but not yet described in published literature, software must be made available to editors/reviewers. We strongly encourage code deposition in a community repository (e.g. GitHub). See the Nature Research guidelines for submitting code & software for further information.

Data

Policy information about availability of data

All manuscripts must include a data availability statement. This statement should provide the following information, where applicable:

- Accession codes, unique identifiers, or web links for publicly available datasets
- A list of figures that have associated raw data
- A description of any restrictions on data availability

The data that support the findings of this study are available from the corresponding authors upon reasonable request.

Field-specific reporting

Please select the one below that is the best fit for your research. If you are not sure, read the appropriate sections before making your selection.

☐ Life sciences ☒ Behavioural & social sciences ☐ Ecological, evolutionary & environmental sciences

For a reference copy of the document with all sections, see nature.com/documents/nr-reporting-summary-flat.pdf

Behavioural & social sciences study design

All studies must disclose on these points even when the disclosure is negative.

Study description	We conducted behavioral, fMRI and rTMS experiments using a rapid sequential button press task under anxiety.
Research sample	82 Participants (33 females) participated in the three experiments. The participants were recruited using a recruiting mailing list
Sampling strategy	We took random sampling approach by sending participants recruiting mailing list maintained by Center for Information and Neural Networks. We chose participants by first come first in strategy.
Data collection	Data was collected by using a touch panel (Experiment 1) and button pad (Experiment 2 and Experiment 3), and stored in a experiment computer. We also recorded physiological data (GSR and PPG) using the BIOPAC system.
Timing	Data was collected between Autumn 2013 and Summer of 2017
Data exclusions	all data collected for the final three experiments were used without omission
Non-participation	non
Randomization	distribution was random

Reporting for specific materials, systems and methods

We require information from authors about some types of materials, experimental systems and methods used in many studies. Here, indicate whether each material, system or method listed is relevant to your study. If you are not sure if a list item applies to your research, read the appropriate section before selecting a response.

Materials & experimental systems

n/a	Involved in the study	
x		Antibodies
x		Eukaryotic cell lines
x		Palaeontology
x		Animals and other organisms
	x	Human research participants
x		Clinical data

Methods

n/a	Involved in the study	
x		ChIP-seq
x		Flow cytometry
	x	MRI-based neuroimaging

Human research participants

Policy information about studies involving human research participants

Population characteristics	Participants were healthy Japanese males (n=49) and females (n=33) aged between 20 and 27.
Recruitment	We sampled the subjects randomly by utilizing the recruiting mailing list maintained by Center for Information and Neural Networks. The experiments were conducted in order (as numbered) and the participants in any experiment were chosen by a first come first participate strategy. For Experiment 2, only those participants who were available on two days (for the behavior and fMRI data collection) were selected . For Experiment 3, we only chose participants who had not experienced TMS before .
Ethics oversight	ethics committee at the Centre for Information and Neural Networks (CINET), Osaka, Japan

Note that full information on the approval of the study protocol must also be provided in the manuscript.

Magnetic resonance imaging

Experimental design

Design type	event-related analysis
Design specifications	20 trials (motor sequences) consisting of 10 button presses. We focused on the start, junction and end of each sequence.
Behavioral performance measures	We collected button presses and inter-press time as well as the number of received electrical shocks. We compared part-learners and single-learners using two-sample t-test.

Acquisition

Imaging type(s)	functional MRI.
Field strength	3 Tesla.
Sequence & imaging parameters	MRI scanning was performed on a Siemens 3T Prisma scanner using an echo planar imaging (EPI) sequence with the following parameters: repetition time (TR) = 3000 ms, echo time (TE) = 25 ms, flip angle = 90°, matrix = 64 × 64, field of view (FOV) = 192 mm, slice thickness = 3 mm, gap = 0 mm, ascending interleaved slice acquisition of 51 axial slices. High-resolution T1-weighted anatomical scans were acquired using an MPRAGE pulse sequence (TR = 2000 ms, TE = 1.98 ms, FOV = 256 mm, image matrix 256 × 256, slice thickness = 1 mm).
Area of acquisition	Whole brain.

Diffusion MRI ☐ Used ☒ Not used

Preprocessing

Preprocessing software	We used SPM12 for all preprocessing.
Normalization	Preprocessing included motion correction, co-registering to the participant's anatomical image, and spatial normalization to the standard Montreal Neurological Institute (MNI) template with a resampled voxel size of 2 mm. Co-registered EPIs were normalized using an anatomical normalization parameter.
Normalization template	East Asian templates provided in SPM12.
Noise and artifact removal	Spatial smoothing was done using a 6-mm Gaussian kernel. Serial autocorrelation was modeled as a first-order autoregressive model, and the data were high-pass filtered at a cutoff of 128 s. We also included 6 dimensional head movement parameters (obtained from realignment) in GLM analysis.
Volume censoring	Brain extraction was done using the segmentation procedures in SPM12.

Statistical modeling & inference

Model type and settings	We conducted univariate analysis based on the standard random-effect model.
Effect(s) tested	We measured the effect of delay at the timing of junction and compared it between part-learners and single-learners.

Specify type of analysis: ☒ Whole brain ☐ ROI-based ☐ Both

Statistic type for inference (See Eklund et al. 2016)	Two-sample t-test based on GLM analysis.
Correction	We adopted FWE correction.

Models & analysis

n/a	Involved in the study
☒	☐ Functional and/or effective connectivity
☒	☐ Graph analysis
☒	☐ Multivariate modeling or predictive analysis
